# Intracranial (structural) changes in obsessive- compulsive disorder: A computerized tomography scan study

**DOI:** 10.4103/0972-6748.62266

**Published:** 2009

**Authors:** Deepak Kumar Giri, Indira Sharma

**Affiliations:** Department of Psychiatry, RINPAS, Kanke, Ranchi, India; 1 Institute of Mental Health, BHU, Varanasi, India

**Keywords:** Computerized tomography scan, Intracranial structural changes, Neuro-imaging, Obsessive-compulsive disorder

## Abstract

**Objective:**

To assess intracranial structural changes in obsessive-compulsive disorder (OCD) with CT scan.

**Materials and Methods:**

Thirty patients of OCD between 21 and 40 years of age and fulfilling various inclusion and exclusion criteria were compared with control group. CT scans of all the patients and controls were taken and compared with regard to ventricular size, Evan’s ratio and ventricular brain ratio (VBR).

**Results:**

Patients of OCD were found to have greater cortical atrophy and scored significantly higher in frontal and parietal area. Only 4 patients had enlarged ventricles, and there were none with a smaller ventricle. Mean Evan’s ratio of patients was lower than that of the control group but the difference was statistically nonsignificant. The mean VBR of patients was lower than that of the control group and the difference was highly significant.

**Conclusions:**

The evidence of neuro-radiological abnormalities in patients with OCD suggests that these disorders should not be considered merely functional in the traditional sense.

Till 1980, obsessive-compulsive disorder (OCD) was thought to be a relatively rare disorder with poor chances of recovery. Current data estimate the lifetime prevalence of OCD to be around 2%-3% of the general population (Behar *et al*., 1984). Organic basis of OCD has been reported by various researchers like Guirdham (1972), Hillbom (1960) and Insel *et al*. (1983). With the advent of successful clinical trials with clomipramine in the treatment of OCD (Karno *et al*., 1988), the search for a primary biochemical abnormality increased, with the focus being on serotonin. The research on the neuroanatomical substrate for OCD has been more sparse and scattered. Current workers laid stress on the role of the basal ganglia (Khanna, 1988; Lensi *et al*., 1996), although some models implicate frontal lobe function (Luxenberg *et al*., 1988; McTavish *et al*., 1999). Insel *et al*. showed ventricular brain ratio (VBR) asymmetry and sulcal prominence measures indistinguishable from those of a matched group of nonpsychiatric control subjects. Rapoport *et al*. (1988) in their study on CT scans showed a mean VBR significantly higher than that of controls and showed spatial perceptual defects similar to those found in patients with frontal lobe lesions. Rasmussen *et al*. (1992) after analyzing brain volumes of 10 male patients found that caudate nucleus volumes in the patients with OCD were significantly less than those of control subjects; but lenticular nucleus, third ventricle and lateral ventricle volumes did not differ between these two groups and no abnormal asymmetry of bilateral structures was detected. In the available studies, there is no consistent evidence of structural etiology in patients with OCD. There is a paucity of literature as far as the CT variables in OCD are concerned, and the Indian scene is especially meager in terms of information available. Considering the fact that some intracranial structural changes may be found in OCD, we planned to carry out this study to find out any intracranial structural changes in patients of OCD.

## MATERIALS AND METHODS

The present study was conducted at the Department of Psychiatry, Institute of Medical Sciences, BHU, Varanasi, from January 2001 to August 2002. The sample consisted of 30 patients of OCD from the outpatient and inpatient sections of the university hospital. Only those who fulfilled the DSM-IV criteria and were between 21 and 40 years of age were included in the study. Patients below 21 or above 40 years, having past history of head injury causing unconsciousness, having history of any significant physical illness, substance abuse and who had used oral steroid medication in the past 3 months prior to onset of psychiatric illness were excluded from the study.

Informed consent was taken from each patient. All 30 patients were interviewed with the help of a pro forma sheet covering identification data, socio-demographic details, precipitating factors, age, total duration of illness, chief complaints, family history, past history of mental illness and pre-morbid personality. Physical and mental illness examinations were carried out in detail and information was recorded. After initial assessment, patients were examined by G.E. Syted 4000i CT scanner; 5-mm contiguous axial sections of brain were obtained from Reid’s baseline to the vertex. The pertinent slices for measurements of VBR and Evan’s ratio and evaluation of sulcal widening for 5 areas were recorded on the imaging film for analysis.

Radiologists blindly rated ventricular size as small, normal or enlarged, and the VBR was estimated by measuring width of lateral ventricle most prominent in the CT slice and the maximum diameter of brain at the same level. It was expressed as percentage.

$$\mathrm{VBR}\;=\;\frac{\mathrm{Width}\;\mathrm{of}\;\mathrm{lateral}\;\mathrm{ventricle}}{\mathrm{Max}.\;\mathrm{of}\;\mathrm{internal}\;\mathrm{skull}\;\mathrm{diameter}\;\mathrm{at}\;\mathrm{the}\;\mathrm{same}\;\mathrm{level}}\;\times \;100$$

Cortical atrophy (sulcal widening) was rated on a 4-point scale for 5 cortical areas (frontal, temporal, parietal, insular and occipital), and a cumulative cortical atrophy score for the separate regions was obtained.

Evan’s ratio was measured as the ratio of the maximum width between the frontal horns of lateral ventricle to maximum internal skull diameter.

$$\mathrm{Evan}’s\;\mathrm{ratio}\;=\;\frac{\mathrm{Max}\;\mathrm{width}\;\mathrm{between}\;\mathrm{the}\;\mathrm{frontal}\;\mathrm{horns}\;\mathrm{of}\;\mathrm{lateral}\;\mathrm{ventricle}}{\mathrm{Max}.\;\mathrm{internal}\;\mathrm{skull}\;\mathrm{diameter}}\;\times \;100$$

Thirty normal CT scans of age- and sex-matched controls were screened and collected to compare the parameters (e.g., cortical atrophy, Evan’s ratio and VBR) with those of OCD patients.

## RESULTS

There were 18 men and 12 women in the study group in comparison to 11 men and 19 women in the control group. Sex distribution did not differ significantly [Table 1]. Majority of the patients in the study group were in the age range of 21-25 years with mean age of 26.73 ± 6.49 years [Table 2]. In the study group, cortical atrophy score between 0 and 2 was found in 16 men and 8 women, and a score between 3 and 5 was found in 2 men and 4 women. These were statistically nonsignificant.

**Table 1:** Sex distribution in study and control groups

	Study group *n* = 30		Control group *n* = 30		χ^2^	*P*
	No.	%	No.	%		
Male	18	60	11	36.7	3.268	>0.05
Female	12	40	19	63.3		

**Table 2:** Age distribution in study and control groups

Age group (in years)	Study group *n* = 30		Control group *n* = 30		χ^2^	*P*
	No.	%	No.	%		
21-25	19	63	14	46.7	4.413	>0.10
26-30	2	7	7	23.3		
31-35	6	20	4	13.3		
36-40	3	10	5	16.6		

The cumulative cortical atrophy score of frontal area of 30 patients in the study group was found to be 12; and in the parietal area, it was 23 — both these values were highly significant in comparison to those of the control group [Table 3].

**Table 3:** Comparisons of cumulative cortical atrophy score in different areas of brain between study and control groups

Cortical atrophy score in different areas of brain	Study group *n* = 30		Control group *n* = 30		χ^2^	*P*
	Cumulative score	Mean ± SD	Cumulative score	Mean ± SD		
Frontal	12	0.4 ± 0.34	27	0.07 ± 0.20	3.07	<0.001[**]
Parietal	23	0.76 ± 0.62	16	0.03 ± 0.12	5.40	<0.001[**]
Insular	3	0.1 ± 0.08	1	0.00 ± 0.00	2.00	>0.05^NS^
Temporal	4	0.166 ± 0.15	2	0.066 ± 0.05	1.24	>0.1^NS^
Occipital	2	0.066 ± 0.05	—	—	1.65	>0.12^NS^

** Highly significant; NS - Not significant

The number of patients in the study group having normal ventricular size was 26; enlarged, 4; and none of them had small ventricular size in comparison to 27, 2 and 1, respectively, in the control group [Table 4].

**Table 4:** Comparison of ventricle size between study and control groups

Ventricle size	Study group *n* = 30		Control group *n* = 30	
	No.	%	No.	%
Normal	26	86.7	27	90
Small	—	—	1	3.3
Enlarged	4	13.3	2	6.7

Mean ventricular size of 19 patients in the 21-25 years age group was 2.64, which was highly significant statistically in comparison with that of control group. The data was also highly significant for age groups 26-30 years and 31-35 years, where ventricle size was 2.43 ± 0.04 and 2.41 ± 0.39, respectively [Table 5].

**Table 5:** Comparison of mean ventricle size between study and control groups

Age group (in years)	Study group *n* = 30		Control group *n* = 30		*t*	*P*
	No.	Mean ± SD	No.	Mean ± SD		
21-25	19	2.64 ± 0.34	14	3.04 ± 0.29	3.53	<0.01[**]
26-30	2	2.43 ± 0.04	7	3.13 ± 0.39	4.37	<0.01[**]
31-35	6	2.41 ± 0.39	4	2.97 ± 0.22	3.11	<0.05[*]
36-40	3	2.73 ± 0.09	5	2.89 ± 0.13	0.84	>0.1^NS^

** Highly significant

* Significant; NS - Not significant

Mean Evan’s ratio was statistically nonsignificant in different age groups [Table 6].

**Table 6:** Comparison of mean evan’s ratio in relation to age between study and control groups

Age group (in years)	Evan’s ratio				*t*	*P*
	Study group (*n* = 30)		Control group (*n* = 30)			
	No.	Mean ± SD	No.	Mean ± SD		
21-25	19	23.21 ± 6.1	14	25.86 ± 1.6	1.81	>0.05^NS^
26-30	2	25.45 ± 0.04	7	25.43 ± 2.3	0.022	>0.1^NS^
31-35	6	25.35 ± 1.61	4	26.50 ± 3.1	0.682	>0.1^NS^
36-40	3	26.7 ± 1.0	5	26.40 ± 2.28	0.255	>0.1^NS^

NS - Not significant

Mean VBR of 22.05 ± 3.11 was also statistically significant in comparison to that of control group [Table 7].

**Table 7:** Comparison of mean ventricular brain ratio between study and control groups

	Study group (*n* = 30)	Control group (*n* = 30)	*t*	*P*
Mean VBR	22.05 ± 3.1	26.00 ± 3.01	4.98	<0.001**

The difference in mean VBR of 21-25 years age group between study group (22.19 ± 3.05) and control group (26.07 ± 2.09) was highly significant. The difference was also highly significant in age group 26-30 years, where mean VBR of study group was 20.30 ± 0.00 and that of control group was 27.43 ± 3.57 [Table 8].

**Table 8:** Comparison of ventricular brain ratio between study and control groups in relation to age

Age group (in years)	Ventricular brain ratio				*t*	*P*
	Study group *n* = 30		Control group *n* = 30			
	No.	Mean ± SD	No.	Mean ± SD		
21-25	19	22.19 ± 3.05	14	26.07 ± 2.09	4.32	,0.01**
26-30	2	20.30 ± 0.00	6	27.43 ± 3.57	4.88	,0.01**
31-35	6	21.55 ± 3.11	5	25.75 ± 1.69	2.837	,0.05*
36-40	3	23.30 ± 2.7	5	24.80 ± 1.29	0.901	.0.05^NS^

## DISCUSSION

With the introduction of CT scan in 1971, revolution suddenly took place in the field of research in psychiatric disorders. In recent years, psychiatrists have employed CT scan as a tool to investigate possible intracranial (structural) changes in brain associated with major psychiatric illnesses.

In the present study, an attempt has been made to assess intracranial (structural) changes in obsessive-compulsive disorder.

The mean age in the study group was 26.76 ± 6.49 years, while it was 28.63 ± 7.99 years in the control group. This finding is consistent with the finding of Schilder (1938). Majority of the patients had duration of illness more than 18 months. For adult patients who came for treatment, OCD appeared to be a chronic condition. This finding is supported by findings of Weissman *et al*. (1994). They reported that 85% of 560 patients in their series had a continuous course with waxing and waning symptom; 10%, a deteriorative course; and 2%, an episodic course. Lensi *et al*. (1996) reported in their study that 65% had chronic course; 26%, episodic course; and 9% had deteriorative course.

The difference in mean cortical atrophy score between study and control groups was found to be statistically nonsignificant. McTavish *et al*. (1999) also showed that VBR asymmetry and sulcal prominence measures in their study were indistinguishable from those of a matched group of nonpsychiatric control subjects.

In the study group, cumulative cortical atrophy score in frontal and parietal areas was highly significant in comparison to that of the control group. A perusal of literature could not reveal any study that stressed atrophy of specific areas of cerebral hemisphere.

The mean ventricle size was statistically highly significant in the age group 21-25 years and was significant in the age group 31-35 years. No definite pattern of association between the duration of illness and ventricular size could be observed. Findings of the present study are in accordance with those of the study by Rapoport *et al*. (1988), in which they found that children with severe primary OCD had higher-than-expected frequency of relative brain ventricular enlargement, and ventricular size did not correlate with sex, onset age or duration of illness.

The mean Evan’s ratio in the study group (24.94 ± 0.52) was smaller than that in the control group (26.00 ± 0.36). However, the difference was statistically nonsignificant. On comparing mean Evan’s ratio between study and control groups in relation to age, findings suggested that in the study group, mean Evan’s ratio was either lower or equal to that of the control group; however, the difference was statistically nonsignificant. On reviewing literature, in any study that had a mention of Evan’s ratio, its relation with duration of illness of OCD could not be found. One study conducted by Behar *et al*. (1984) reported that when Evan’s ratios of patients and controls were compared, a significant difference was found between patients (mean ± SD, 0.31 ± 0.3) and controls (mean ± SD, 0.27 ±0.05).

The mean VBR in patients showed a continuous increase as the duration of illness increased. This finding suggested that as the duration of illness increases, the VBR also increases in OCD. The findings are inconsistent with the findings from the study by Behar *et al*. (1984). They showed that patients had a mean VBR significantly higher than that of the controls and showed spatial-perceptual deficits similar to those found in patients with frontal lobe lesions.

When the results of the present study were compared with those of normal CT scans of controls, intracranial structural changes were found particularly in VBR, ventricular enlargement and Evan’s ratio. Observations about some aspects in this study could not be compared with those of other studies because none of them have mentioned about these aspects of the present study. Correlation of these changes may throw light on etiological significance, course and prognosis.

## CONCLUSION

The evidence of neuro-radiological abnormalities in patients with obsessive-compulsive disorders suggests that these disorders should not be considered merely functional in the traditional sense. An important task for researchers is to find out all specific structural abnormalities associated with these conditions and try to correlate them with various important variables like age, duration of illness, clinical presentation and prognosis.

## References

[CIT1] Behar D, Rapoport J. L, Berg C.J, Denckla M. B, Mann L, Cox C (1984). Computerized Tomography and neuropsychological test measures in adolescents with obsessivecompulsive disorder. Am J Psychiatry.

[CIT2] Guirdham A (1972). Obsession.

[CIT3] Hillbom E (1960). After effects of brain injuries. Acta Psychiatr Neurologica Scandinavia, Suplementum.

[CIT4] Insel T. R, Donnelly E. F, Lalakea M. L, Alterman I. S, Murphy D. L (1983). Neurological and neuropsychological studies of patients with obsessive-compulsive disorder. Biological Psychiatry.

[CIT5] Karno M, Golding J. M, Sorenson S. B, Burnam M. A (1988). The epidemiology of obsessive-compulsive disorder in five US communities. Arch Gen Psychiatry.

[CIT6] Khanna S (1988). Obsessive-compulsive disorder: Is there a frontal lobe dysfunction?. Biological Psychiatry.

[CIT7] Lensi P, Cassano G. B, Correddu G, Ravagli S, Kunovac J. L (1996). Obsessive-compulsive disorder: Familialdevelopmental history, symptomatology, co morbidity and course with special references to gender related differences. Br J Psychiatry.

[CIT8] Luxenberg J. S, Swedo S. E, Flament M. F, Friedland R. P, Rapoport J, Rapoport S. I (1988). Neuroanatomic abnormalities in obsessive-compulsive disorder detected with quantitative X-ray computed tomography. Am J Psychiatry.

[CIT9] McTavish D, Benfield P (1999). Clomipramine: An overview of its pharmacological properties and a review of its therapeutic use in obsessive-compulsive disorder and panic disorder. Drugs.

[CIT10] Rapoport J. L, Wise S. P (1988). Obsessivecompulsive disorder: Evidence for basal ganglia dysfunction. Psychopharmacology Bulletin.

[CIT11] Rasmussen S. A, Eisen J. L (1992). The epidemiology and clinical features of obsessive-compulsive disorder. Psychiatric Clinics of North America.

[CIT12] Schilder P (1938). The organic background of obsessions and compulsions. Am J Psychiatry.

[CIT13] Weissman M. M, Bland R. C, Canino G. J, Greenwald S, Hwu H. G, Lee , C.K (1994). The cross-national epidemiology of obsessive-compulsive disorder. The crossnational collaborative group. J Clin Psychiatry.

